# Using seemingly unnecessary illustrations to improve the diagnostic usefulness of descriptions in taxonomy–a case study on *Perochaeta orientalis* (Diptera, Sepsidae)

**DOI:** 10.3897/zookeys.355.6013

**Published:** 2013-11-25

**Authors:** Yuchen Ang, Ling Jing Wong, Rudolf Meier

**Affiliations:** 1 Evolutionary Biology Laboratory, Department of Biological Sciences, National University of Singapore, 14 Science Drive 4, Singapore 117543, Singapore

**Keywords:** Taxonomy, species descriptions, illustrations, bioimaging, videography, Sepsidae

## Abstract

Many species descriptions, especially older ones, consist mostly of text and have few illustrations. Only the most conspicuous morphological features needed for species diagnosis and delimitation at the time of description are illustrated. Such descriptions can quickly become inadequate when new species or characters are discovered. We propose that descriptions should become more data-rich by presenting a large amount of images and illustrations to cover as much morphology as possible; these descriptions are more likely to remain adequate over time because their large amounts of visual data could capture character systems that may become important in the future. Such an approach can now be quickly and easily achieved given that high-quality digital photography is readily available. Here, we re-describe the sepsid fly *Perochaeta orientalis* (de Meijere 1913) (Diptera, Sepsidae) which has suffered from inadequate descriptions in the past, and use photomicrography, scanning electron microscopy and videography to document its external morphology and mating behaviour. All images and videos are embedded within the electronic publication. We discuss briefly benefits and problems with our approach.

## Introduction

Many species descriptions–especially older ones–are very brief: they comprise of discussions and illustrations of diagnostic morphology, geographical distribution, and only occasionally some biology (e.g., see Appendix). The morphology sections are often limited to the most conspicuous features that can be used to differentiate and identify the target species from other species known to the scientific community at the time of description. In the past, this minimalist approach was necessary because journals had tight page restrictions and the cost of including many illustrations was high; this was a particularly serious problem for colour and halftone illustrations. Their high cost contributed to the widespread use of line-drawings in descriptive papers. However, such an exiguous approach towards descriptions is no longer needed given that these restrictions have largely disappeared. While line drawings remain important for clearly illustrating diagnostic features, a description can now afford to include more and different types of data. Electronic journals have fewer limitations on page numbers, and taxonomists now have ready access to high-resolution photography (Ang et al. 2008a) or even µ-CT (Schneeberg et al. 2012), allowing large amounts of data to be acquired quickly. Furthermore, halftone and colour illustrations do not incur additional cost in most electronic publications, and even videos can be embedded in electronic publications, so that primary evidence on the biology of a species can be included (van Achterberg and Durán 2011).

Embracing these new opportunities has many advantages. One is that more data makes it less likely that today’s descriptions will be inadequate in the future: a large number of images may serendipitously capture features that will only be revealed to be important in the future. This does not distract from the importance of line drawings, which have the advantage of highlighting important features and can accommodate intraspecific variability (see Discussion). However, line-drawings have the disadvantage that they are unlikely to capture character systems of future importance. For example, 19^th^ and some early 20^th^ century entomologists did not anticipate the importance of genitalia and microtrichosity (pruinosity) patterns in species identification they remained undescribed. Had current-day imaging techniques been available and used by these taxonomists, genitalia [at least “claspers” (hypopygia)] and microtrichosity data would have been captured despite their perceived unimportance at the time of description.

Employing these imaging techniques can also protect against bad taxonomy. For example, Francis Walker (1809–1874), while one of the most prolific taxonomist of his time, was also well known for his poor-quality judgement and descriptions that resulted in numerous synonyms [as his obituary laments; ‘*More than twenty years too late for his scientific reputation, and after having done an amount of injury almost inconceivable in its immensity, Francis Walker has passed from among us*’ (Carrington 1874)]. If the inclusion of large numbers of illustrations and images had been the taxonomic standard in descriptions published in the 19^th^ century, many of his “new species” would not have been published and/or it would have been easier to resolve the taxonomic problems that were caused by his work.

The use of modern imaging is slowly beginning to gain traction in taxonomy (Neusser et al. 2011) because digital images are particularly suitable for dissemination of taxonomic knowledge over the internet. Museum specimen trays can now be accessed virtually (Schmidt et al. 2012), digital reference collections in the form of high-resolution images can be assembled (Ang et al. 2013) and easily curated and updated on wiki sites (Hendrich and Balke 2011). Furthermore, videos, 3D models and other large datasets can be embedded in PDF files or at least linked as supplementary data (Faulwetter et al. 2013). This is advantageous because it encourages the sharing of many kinds of data (e.g., morphology, behaviour, DNA sequences) which can provide different perspectives on difficult taxonomic issues such as cryptic species complexes (Tan et al. 2010).

Here, we present a re-description of *Perochaeta orientalis* (de Meijere 1913) (Sepsidae: Diptera) consisting of morphological, behavioural, DNA sequence, biogeographical, and biological data. We re-describe the species, include comprehensive external morphology data by imaging all views of male and female specimens, and describe their mating behaviour profile along with video data. The re-description of *Perochaeta orientalis* is warranted because the two existing treatments (de Meijere 1913, Duda 1926) are both inadequate for reliable species identification. In addition, both are written in German and published in discontinued publications, which limits their accessibility.

*Perochaeta* is a small Oriental genus, with currently six described species (Ang and Meier 2010, Iwasa and Thinh 2012). This includes *Perochaeta orientalis*, *Perochaeta cuirassa* Ang, 2010, *Perochaeta dikowi* Ang et al., 2008, *Perochaeta exilis* Iwasa, 2011, *Perochaeta hennigi* Ozerov, 1992 and *Perochaeta lobo* Ang, 2010. In order to facilitate the identification of all described species in the genus, we also provide diagnostic differences between all species.

## Materials and methods

*Collection and rearing of specimens*. All new material was acquired from a laboratory culture. This culture was established based on a single female adult specimen collected from a mid-elevation site in Malaysia (Cameron Highlands, 1600m ASL) and reared based on methods described in Ang et al. (2008b). For mating experiments, adult males and females were separated within a day of emergence to obtain virgin flies. These flies were allowed to sexually mature for three days post-eclosion before mating trials began. Specimens (in 70% ethanol) used in this re-description are kept in the Raffles Museum of Biodiversity and Research (RMBR), National University of Singapore, Singapore.

**Figure 1.**
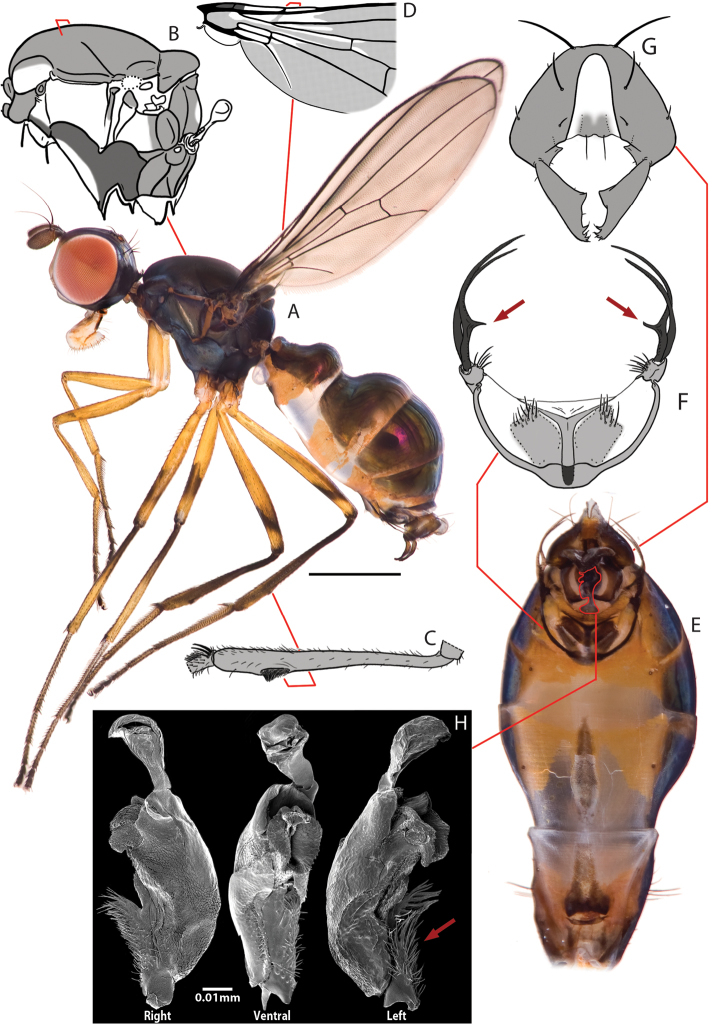
Key views and structures of *Perochaeta orientalis*, Male. **A** Habitus, lateral view **B** Pleural microtomensity pattern; (white = smooth, light grey = lightly microtomentose, dark grey = heavily microtomentose) **C** Rear tibia, with focus on osomterium **D** Basal section of wing showing microtrichosity pattern (white=smooth, light grey=with microtrichia) **E** Whole abdomen, ventral view **F** Sternite appendage **G** Hypopygium, dorsal view **H** Phallus, right, ventral and left views; red arrow indicates basal spiny flap. Scale bars = 0.5mm unless otherwise stated.

**Figure 2.**
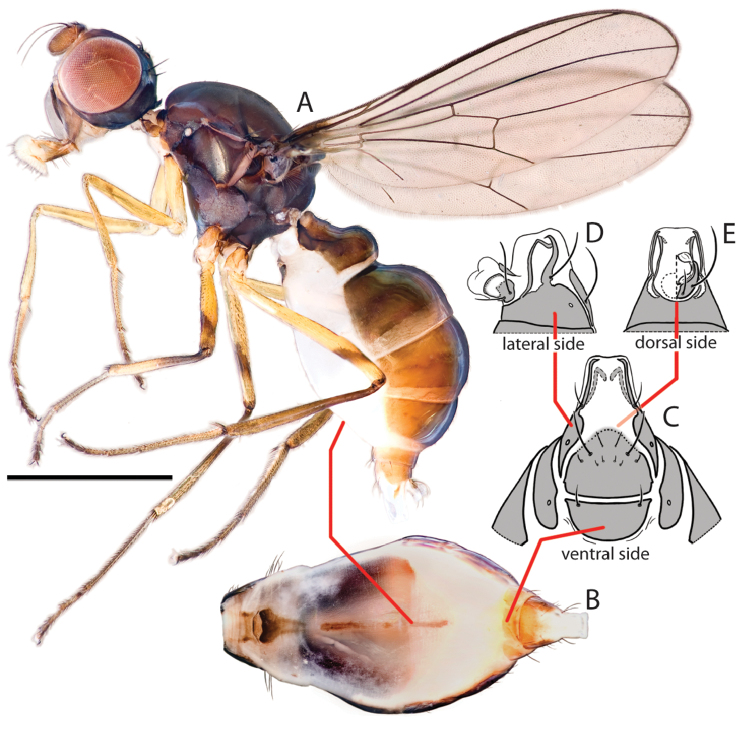
Key views and structures of *Perochaeta orientalis*, Female. **A** Habitus, lateral view **B** Whole abdomen, ventral view **C** Abdominal posterior, ventral view **D** Same, lateral view **E** Same, dorsal view. Scale bar = 0.5mm.

**Figure 3.**
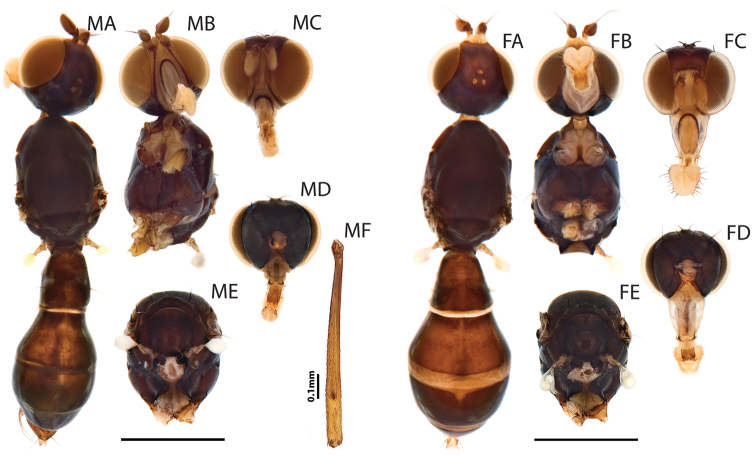
Additional views for *Perochaeta orientalis*, Male (MA-MF) and Female (FA-FE). M and F prefixes refer to male and female specimen respectively. **A** Habitus, dorsal view (sans wings) **B** head and thorax, ventral view **C** Head capsule, anterior view **D** Head capsule, posterior view **E** Thorax, posterior view **F** (male only)–Rear tibia, dorsal view showing osmeterium. Scale bars = 0.5mm unless otherwise stated.

**Figure 4.**
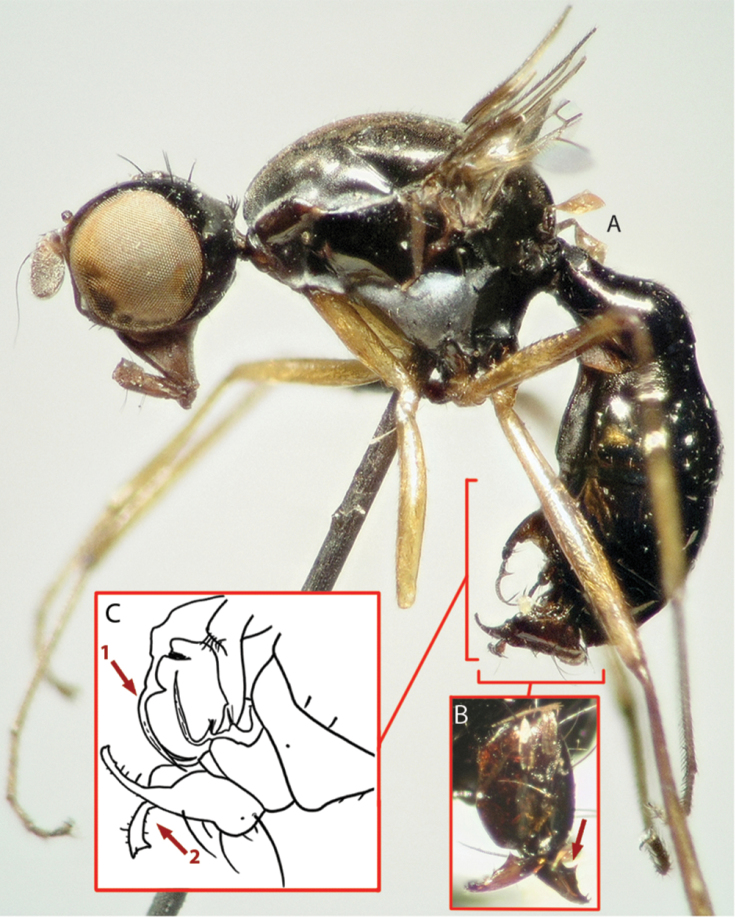
Images of holotype (**A, B**) and drawing (**C**) from description for *Perochaeta orientalis*, male. **A** Image of habitus, lateral view **B** Image of hypopygium, dorsal view; red arrow pointing to the median protrusion on the surstylus **C** Drawing of abdominal posterior (lateral view) as reproduced from Duda (1926); red arrow 1 shows how illustration has fused the two setae into one, red arrow 2 shows how the drawing fails to display the median protrusion as seen in Fig. 1G.

*Photography & illustrations*. Male and female specimens were extracted from the culture for re-description. The habitus for both sexes were imaged using the Visionary Digital^TM^ Plus Lab System (CF4P3 magnification). Several other structures were also imaged and then digitally transferred into line drawings through tracing with a Wacom® PTZ 630 tablet in Adobe® Photoshop® CS4. Images and illustrations of important diagnostic features are shown in Figs 1 and 2, while images for additional views are shown in Fig. 3. Images of the holotype (Fig. 4A, B) were provided by the Hungarian Natural History Museum, Budapest, Hungary.

*Scanning electron microscopy (SEM)*. A phallus was dissected and dehydrated in an alcohol series, then critical-point dried with CO2 (Balzers® CPD-030) and mounted on a metal stub and platinum sputter-coated (JEOL® JFC 1600 Pt Fine Coater). SEM was performed at 100× with the JEOL JSM 6510 SEM. The image was then cleaned up with Adobe® Photoshop® CS4, and incorporated into Fig. 1.

*Mating experiments*. Each mating trial involved two male-female pairs because this species has very low mating success rates. The flies were introduced simultaneously into a small petri-dish and placed under a Leica MZ16A microscope. The mating behaviour was then recorded with an analogue video recorder (36 trials). Recording of behaviour began immediately upon the introduction of specimens into the petri-dish, and ended after 45 minutes if no mounting attempts made, or if they were not successful. The recordings were afterwards digitised and the non-linear editing software Final Cut Pro was used to study the behaviour ‘frame by frame’ (25 f.p.s.) in order to create a detailed mating profile. This profile was then compared with that of *Perochaeta dikowi* (Ang et al. 2008b). Video clips of relevant behaviours were extracted and put together with Windows Movie Maker (2012 ver.), and embedded as a video object in PDF using Adobe® Acrobat® Pro X.

*Online curation of specimens*. All images, videos and the appendix in their original resolution are deposited in the species entry for Sepsidnet, an online digital reference collection dedicated to the Sepsidae of the world. These materials are also deposited as a project in Morphobank (Project 1062).

*Taxonomic terminology*. We adopt the terminology as described by Merz and Haenni (2000) for adult non-terminalia morphology and Sinclair (2000) for genitalia.

## Taxonomy and behaviour

### Perochaeta orientalis

(de Meijere, 1913)

http://species-id.net/wiki/Perochaeta_orientalis

Figs 1 –6

#### Material examined.

*Holotype* ♂ (Figs 4A, B). Type locality: “Chip Chip” (Jiji, = 集集) Township, Nantou County (南投), Taiwan ROC [likely, approximate coordinates 23°50'7"N, 120°46'4"E] (type label info: “Formosa Sauter. Chip-Chip 909. III. *Nemopoda orientalis* det de Meijere. Type.”). ♂ in the Hungarian Natural History Museum, Budapest, Hungary.

#### Additional material

(Figs 1–3). Locality: Brinchang Jungle Trail, Cameron Highlands, Pahang, Peninsular Malaysia [4°30'9.55"N, 101°23'20.85"E. 1600m ASL]. Isoline culture based on ♀ collected 4.I.2011 (R. Meier). ♂♂♀♀ in the Raffles Museum of Biodiversity Research.

#### Morphological diagnosis (adult).

**Figure 5.**
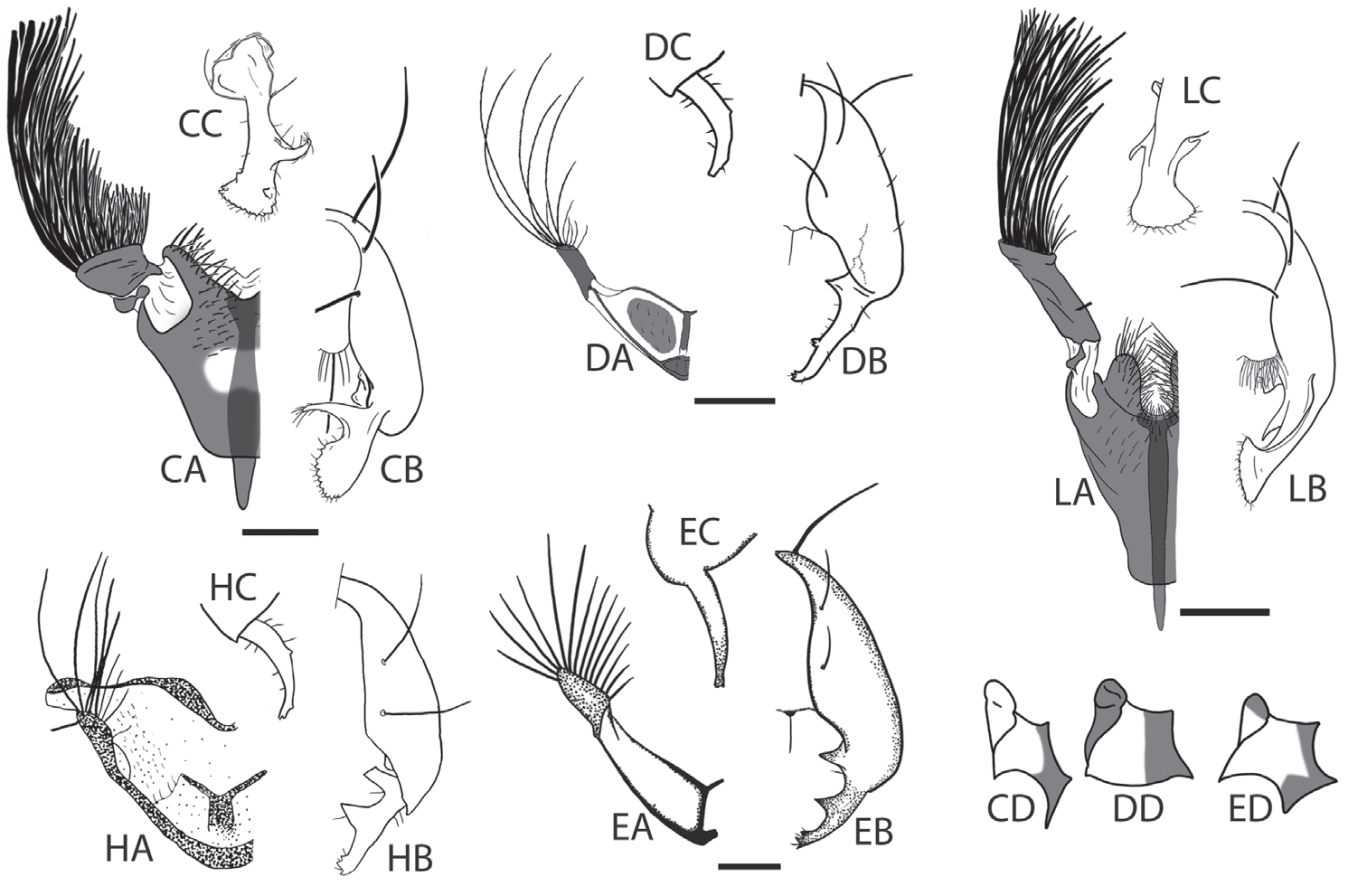
Hypopygia, sternite appendages and anepimeral + greater ampullal microtrichosity of the five other *Perochaeta*: *Perochaeta cuirassa* (CA-CC), *Perochaeta dikowi* (DA-DC), *Perochaeta exilis* (EA-EC), *Perochaeta hennigi* (HA-HC) and *Perochaeta lobo* (LA-LC); adapted from Ang and Meier (2008; *Perochaeta cuirassa* and *Perochaeta lobo*), Ang et al. (2008; *Perochaeta dikowi*), Iwasa (2011; *Perochaeta exilis*) and Ozerov (1992; *Perochaeta hennigi*). Suffixes refer to: **A** sternite appendage, left side ventral view **B** hypopygium, right side dorsal view **C** Surstylus, lateral view **D** Anepimeron + greater ampulla [image not available for *Perochaeta hennigi* (prefix H)]. *Perochaeta lobo* (prefix L) has a similar anepimeral microtrichosity as *Perochaeta cuirassa* (CD). Scale bars = 0.5mm.

Male *Perochaeta orientalis* are most easily differentiated from other described *Perochaeta* species based on two large, flattened bristles of the main tuft on the sternite appendage, of which one has a triangular, submedial protrusion (red arrows on Fig. 1F) while all other described *Perochaeta* species have unmodified bristles (Figs 5 with suffix ‘A’). The surstylus in *Perochaeta orientalis* (Fig. 1G) is also unique in that the median inward protrusion consists of a large, broad-based triangle that spans a third of the surstylus (see Figs 5 with suffixes ‘B’ and ‘C’). The hind tibia of *Perochaeta orientalis* also has a distinct, raised osmeterium (Fig. 1C) which is barely visible or missing in other *Perochaeta*. Adult female *Perochaeta orientalis* can be distinguished from the females of *Perochaeta dikowi* (the only other species with a female description) based on the presence of sternites 3 and 4 (Fig. 2B), which are missing in the latter. For both sexes, the pleural, thoracical microtrichosity for *Perochaeta orientalis* (red arrow on Fig. 1B) is most similar to that of *Perochaeta exilis* (Fig. 5ED) because it is tomentose on the posterior third of the anepimeron and the dorsal tip of the greater ampulla. In contrast, *Perochaeta cuirassa* and *Perochaeta lobo* (Fig. 5CD) have a glossy greater ampulla, while *Perochaeta dikowi* is pruinose wholly on the greater ampulla and on slightly less than the posterior half of the anepimeron (Fig. 5DD).

#### Morphological description.

*Colour*. Similar in males (Fig. 1A) and females (Fig. 2A). Head capsule black except for face and a connecting thin strip below the eye, which is light-brown. Antennal pedicel dark brown, first flagellomere paler. Proboscis dark-brown with yellow labellum. Thorax wholly black, abdomen with glossy dark-brown tergites and sternites. All femora largely yellow with diffuse obfuscate rings post medially (faint on fore femur). Fore tibia wholly yellow; mid tibia darkened on the basal half; rear tibia entirely dark. All tarsi with first two segments yellow and last three dark-brown. Wing cells clear except for darkened basicostal cell and basal third of costal cell. Veins mostly dark brown. Calypter creamy; haltere whitish with brown base.

*Head*. Similar in males and females (Figs 1A, 2A). Roundish; facial carina short and shallow, facial area receding. Gena and parafacial region narrow. Ocellar prominence and occipital region lightly microtomentose. Chaetotaxy: *ocellar* longer than divergent *postocellar*; 1 *outer vertical*; *inner vertical* absent; *orbital* very reduced; 2 *vibrissae*; 2–3 weak *postoculars*; Lower fascial margin lined with setulae.

*Thorax*. Similar in males and females. Scutum, scutellum and subscutellum lightly microtomentose. Mediotergite microtomentose but glossy in the medial region (Figs 3ME, 3FE). Scutellum twice as wide as long (Figs 3MA, 3FA). Pleural pruinosity pattern (Fig. 1B): Protonotopleural lobe glossy on pleural region but microtomentose on dorsal region. Proepisternum fully microtomentose. Anepisternum largely glossy with anterioventral region densely microtomentose. Katepisternum with dense tomentosity except for glossy anterioventral region. Greater ampulla lightly microtomentose on the dorsal tip. Anepimeron glossy with lightly microtomentose strip on posterioventral region. Katatergite, katepimeron, metakatepisterum, meron and metepimeron lightly-dusted. Chaetotaxy: 1 *apical scutellar*, 1 reduced, setulae-like *basal scutellar*, 1 *dorsocentral*, 1 *postalar*, 1 *supraalar*, 2 *notopleural*, 1 *postpronotal*, 1 *anepisternal* and 1 *posterior spiracular*. Postpronotoum, prescutum and anepisternum with few, sporadic setulae.

*Legs*. Fore legs unmodified in males and females; all femora and tibiae without robust setae except for a longitudinal row of short spines on the anterior basal half of mid femur. Male rear tibia with a small but distinct osmeterium with raised hairs at the posteriodorsal region, and with three enlarged ventral setae on basitarsus (Fig. 1C). Females similar but lacking in osmeterium.

*Wings*. Similar in males and females. Slender. Without apical pterostigma. Veins bare. Wing microtrichia pattern (basal half; Fig. 1D): cells covered with microtrichiae except for subcostal, basal-medial, posterior-cubital cells and alula. Costal, radial 1, radial 2+3, radial 4+5, basal-radial, disco-medial, anterior cubital cells and anal lobe with portions lacking microtrichia. Radial-medial cross-vein divides discal-medial cell by ratio of 2: 1. Length: 4.4–4.8 mm.

*Male abdomen*. Ventral view (Figs 1E, F). Syntergite 1+2 to tergite 5 normal, tergite 6 missing, syntergite 7+8 present and extending ventrad as a narrow sclerite. Spiracles 1–4 on intersegmental membrane, spiracle 5 on ventral margin of tergite 5, spiracle 7 and 8 adjacent on margin of syntergite 7+8. Sternite 1 as a thin lateral band with tapering ends while sternite 2 is triangular, tapering posteriorly; sternite 3 is longitudinally oblong. Sternite 4 heavily modified into paired moveable appendages [Fig. 1F; see Bowsher et al. (2013) for a discussion on the evolution of the appendages and Fig. 5 for sternite appendage illustrations of other *Perochaeta*]: largely desclerotized except for anterior margin as well as two rectangular regions laterally off the median. Two stout moveable appendages branch off laterally, each with a tuft of small short bristles facing the inner side of the sternite and two large, flattened and inward-curving bristles on the apices, of which one is pinched sub medially, resulting in a tooth like furcation on the inward side (red arrows on Fig. 1F).

*Hypopygium* (Fig. 1G). Cercal plate with two very weak lobes, each with one setae. Hypopygium triangular with a large tooth-like projection originating from the inner base of the surstylus. Surstylus itself fused to hypopygium and branches off dorsally. Each surstylus is curved ventrally, with a large, flattened, inward-facing posteriomedial triangular process; terminus with “teeth” and setulae.

*Phallus* (Fig. 1H). Basal region with scales on left side and relatively smooth on right side (crinkles and cracks on the surface are artifacts due to drying process). Basal region with large flap adorned with numerous long spines. Distal portion short (ca. 1/3 of basal portion) and membranous. We refrain from assigning terminology, for reasons explained in Discussion.

*Female abdomen* (Fig. 2B–E). Syntergite 1+2–tergite 5 similar to male, tergites 6 and 7 well defined and sclerotized. Spiracles 1–5 in intersegmental membrane while spiracles 6 and 7 are within the tergites. Sternites 1 and 2 similar to male, sternite 3 as a very thin longitudinal strip. Sternite 4 also a thin strip with barely visible sclerotization and a diffuse margin, sternite 5 missing. Sternites 6 as a lateral rectangle and sternite 7 tapering posteriorly. Postabdominal segments 6 and 7 with the tergites and sternites separated laterally, the sternites (like the tergites) thus very broad and short; segment 8, when not invaginated, long, extended posteriorly and ventrally, with a ventral element (sternite 8) on each side that remains separated at tip and a dorsal element (tergite 8) that forms the usual pair of ring-like bars that do not quite touch apically. Cercus small and round, with hypoproct present, bare.

#### Mating behaviour.

**Figure 6.**
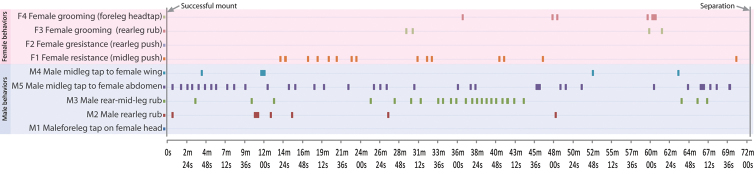
Copulatory profile for *Perochaeta orientalis*, as described in Section 2 (Copulation). Horizontal bars in graph indicate point in time (X-axis) where then the particular behaviour (Y-axis) is performed. The profile begins from when the male mounts the female, and ends when they begin to separate (total time = 72m 30s).

Here, we conducted 36 mating trials with virgin males and females. Only two of these trials were successful (=5.6% mating success rate), and the copulation time for these two were ca. 75 and 72 minutes. Virgin mating behaviour can be divided into four phases: (1) courtship, (2) approach and mount, (3) copulation and (4) separation. The copulatory profile (section 3) for *Perochaeta orientalis* is shown in Fig. 6, based on a frame-by-frame analysis of one of the trials and documented in Video 1 (time in video given as mm:ss). Where available, we will compare and differentiate the behaviour of *Perochaeta orientalis* with *Perochaeta dikowi* (Ang et al. 2008b) which is the only other *Perochaeta* species with a known mating profile. Our efforts to provide detailed mating behaviour for *Perochaeta orientalis* is part of a larger series of papers investigating of mating behaviour in sepsids (e.g., Ang et al. 2008b, Puniamoorthy et al. 2008, Puniamoorthy et al. 2009, Tan et al. 2010, Tan et al. 2011). As discussed in Puniamoorthy et al. (2009), attention to detail is important because sepsid mating behaviour is apparently species-specific.

**Video 1.** Video montage for the various behaviours described. Section 1, Courtship: Male wing-flutter dance (00:07). Section 2, Approach and Mount: Failed attempt with female resistance, lateral view (00:15), Successful mount, dorsal view (00:29). Section 3, Copulation: M1 Male fore leg tap to female head (00:41), M2 Male rear leg rub (01:03), M3 Male rear- to mid-leg rub (01:10), M4 Male mid legs tap to female wing (01:18), M5 Male mid legs tap to female abdomen (01:29), F1 Female resistance (mid legs push) (01:39), F2 Female resistance (rear leg push) (01:51), F3 Female grooming (rear leg rub) (02:00), F4 Female grooming (fore leg-head rub) (02:06). Section 4, Separation (02:15). Video available for download in full resolution from http://www.pensoft.net/J_FILES/1/articles/6013/Ang_Wong_Meier_Video_1.avi

*Courtship*. When the male detects and shows interest in a female, he courts the female by using a “wing flutter dance”; i.e., he rapidly circles the female from his side while fluttering the wing facing the female (00:07). This behaviour is not observed in *Perochaeta dikowi*.

*Approach and mount*. The male will approach the female from the rear and attempt to mount her. Unlike most sepsid species, *Perochaeta orientalis* males lack modified fore legs, and do not clasp the female wing or perform pre-copulatory behaviours when mounted like other sepsids (Puniamoorthy et al. 2008). Instead, he mounts similarly to *Perochaeta dikowi*; using his fore tarsi to hold on to the female’s abdomen whilst bending his abdomen forward. He then extends his sternite brush to contact the genital region, while the surstylus attempts to clasp the female genitalia (00:15 & 00:29). A crucial difference between the two species is that *Perochaeta dikowi* uses his sternite brush to contact the anterior portion of the female abdomen before sliding towards her posterior, while *Perochaeta orientalis* immediately contacts the genital region (see attempt in 00:15). At this stage, females show strong rejection behaviour towards the males which explains the low mating success rate. Males are kicked with mid- and hindlegs and/or the abdomen is raised to prevent genital contact (00:15). All resisting females remained unmated and only those males succeeded in mating that encountered willing females (00:29). In *Perochaeta dikowi*, female resistance is much lower and mating success rates were 28.6%.

*Copulation* (Fig. 6). Once the male locks its genitalia with the female, they copulate for a long time (73.7±1.2 min; based on the two successful trials), which is over 3 times longer than that in *Perochaeta dikowi* (22.6±2.48 min). There are periods of rest and activity during copulation. During rest, males place their fore tarsi on the female pronotal callus while mid- and rear legs are splayed out. During active periods, the male displays five types of behaviours: “M1: fore leg head tap”–males using fore tarsi to tap repeatedly on female head (00:41), “M2: rear leg rub”–males rubbing rear legs together (01:03), “M3: rear-mid-leg rub”–males rubbing rear legs with mid legs (01:10), “M4: mid legs wing tap”–males using mid legs to tap repeatedly on female wing (01:18) and “M5: mid legs abdomen tap”–males use mid legs to tap repeatedly on female abdomen (01:29). Behaviours M3 and M4 mostly occur after M1 and M2, suggesting a transfer of substance from the rear tibial osmoteria to the mid legs and then onto the female wing and/or abdomen. Female resistance was recorded even after copulation commenced; the female mostly used her mid legs (F1; 01:39) and only occasionally her hindlegs to push against the male (F2; 01:51). The female also indulged in grooming herself at times, either performing a rear leg rub (F3; 02:00) or a fore leg-head rub (F4; 02:06)

*Separation*. Just prior to separation, the male performs the “fore leg head tap” as well as the consecutive “rear-mid-leg rub” and “mid legs abdomen tap”. The separation event itself is initiated by the male, where he turns 180° and pulls away from the female (02:15). Both males and females will also use their rear legs to push against each other during this time. This is similar in *Perochaeta dikowi*.

#### Distribution, laboratory records and DNA sequence information.

*Biogeography*. *Perochaeta* has been consistently found only in mid- to high-elevation areas [see Ang and Meier (2010) for a discussion on the genus’s biogeographical distribution]. *Perochaeta orientalis* itself was first collected by Sauter from two township localities in the central highlands (Nantou County; = 南投縣) of Taiwan: Jiji (“Chip Chip”, = 集集) and Puli (“Polisha”, = 埔里; approximate coordinates 23°57’56”N, 120°57’57”E) (de Meijere 1913). While the elevation of these two townships are relatively low (ca. 300m for Jiji and 500m for Puli), they are both immediately enclosed by mountain ranges that reach to excesses of 2500m. Specimen collection in Sauter’s expedition would likely be from these mountainous regions. It is thus possible that *Perochaeta orientalis*–like its other congeners in *Perochaeta*–is a higher-elevation specialist limited to the hills and mountains of the Oriental region. It has been recorded in Taiwan, Indonesia (Sulawesi I.), East and West Malaysia, as well as the Philippines (Luzon I., Mindanao I.) (Ozerov 2005).

*Laboratory records*. Under laboratory conditions, *Perochaeta orientalis* has been bred successfully from bovine (cow and gaur) dung. They are also attracted to this substrate in the wild, which makes sampling an area for *Perochaeta* a “bait-and-wait” strategy.

*DNA sequence information.* Molecular data from our new *Perochaeta orientalis* material are presented as part of the updated sepsid phylogeny (Lei et al. 2013). Nine mitochondrial and nuclear genes are sequenced and uploaded to Genbank. Their accession numbers are: 12S - KF199478, 16S - KF199525, COII - KF199667, COI - KF199842, CYTB - KF199714, 18S - KF199572, 28S - KF199618, ATS - KF199795, H3 - KF199739. Genetic distances for COI between existing species with DNA records (*Perochaeta cuirassa*, *Perochaeta dikowi* and *Perochaeta lobo*) were calculated using SpeciesIdentifier (Meier et al. 2006). *Perochaeta orientalis* has the most similar sequence to *Perochaeta dikowi* (3.82%; Table 1), a distance that is well in excess of what is normally found between dipteran species (Meier et al. 2008).

**Table 1.** A summary of the pairwise distances between the COI of *Perochaeta orientalis* with that of *Perochaeta cuirassa* (KF199839), *Perochaeta dikowi* (KF199840) and *Perochaeta lobo* (KF199841). *Perochaeta orientalis* has the most similar sequence to *Perochaeta dikowi*’s (3.82%), and all pairwise distances are relatively high.

	*Perochaeta orientalis*	*Perochaeta cuirassa*	*Perochaeta dikowi*	*Perochaeta lobo*
***Perochaeta orientalis***	0.00%	11.44%	**3.82%**	13.15%
*Perochaeta cuirassa*	8.70%	0.00%	12.95%	11.89%
*Perochaeta dikowi*	11.89%	12.95%	0.00%	8.70%
*Perochaeta lobo*	13.15%	**3.82%**	11.44%	0.00%

## Discussion

### Concordance with precedent descriptions and holotype

The decision to re-describe *Perochaeta orientalis* was based on the quality and accessibility of the two precedent descriptions by de Meijere and Duda (Appendix). de Meijere’s description (1913) was a short paragraph written in German, devoid of illustrations, and published in a journal that has been discontinued; i.e., it was a good case for a relatively inaccessible description that was also insufficient for reliable species identification. Duda’s re-description (1926) was much more detailed, but only one illustration was presented which lacked clarity (Fig. 4C): For example, while it did show the two long, flattened setae found in *Perochaeta orientalis*, they were drawn fused at the base as a single bifurcated seta (Arrow 1). The hypopygium was also drawn in such a way that it failed to illustrate the large median triangular protrusion on the surstylus (Arrow 2; cf. Fig. 1G). In this case, much effort went into text instead of illustrations, which still resulted in an unclear species diagnosis. It was only through re-imaging of the holotype specimen (Fig. 4A, B) that we were able to determine that our material was indeed *Perochaeta orientalis*, based on the lateral thoracical microtrichosity pattern (c.f. Figs 1A, 4A), the bristle morphology on the sternite 4 appendage (square parenthesis on Fig. 4A) as well as the large median protrusion on the surstylus (arrow on Fig. 4B). Overall, there is no doubt that a photograph of the holotype would have been much more informative than the line drawing in Duda (1926).

### The phallus as anticipatory data

**Figure 7.**
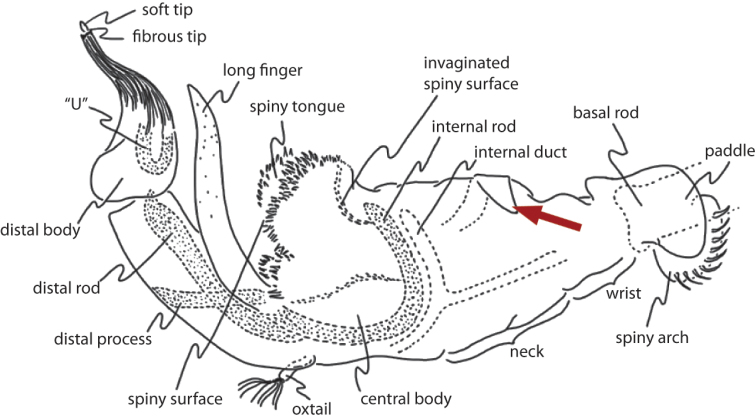
Illustration of *Archisepsis* phallus, as reproduced from Eberhard and Huber (1998). Red arrow indicates region that may be homologous to the basal spiny flap in *Perochaeta orientalis*.

In this paper we include images of the unlabelled phallus (Fig. 1H). There is still a dearth of information on this structure in Sepsidae, but we anticipate that it will gain in importance in the future. It is well-recognised that insect genitalia evolve rapidly and divergently, and are often the most reliable source of characters to delimit and describe species (Eberhard 1985). However, the phallus is almost never described in Sepsidae because species identification can usually be accomplished using the more exposed genitalia (e.g. hypopygium) and other secondary sexual characters [e.g. fore legs and modified sternites; see Pont and Meier (2002)] while the phallus is often inaccessible and requires dissection and slide preparation. A review of current literature revealed that only two publications included detailed information on sepsid phallus (‘aedeagus’) and the authors either refrained from a description in the text (Zuska 1965) or used informal terms such as ‘ spiny tongue’, ‘long finger’ and ‘oxtail’ (Fig. 7; Eberhard and Huber 1998). Unfortunately, the phallus is very variable between species and genera, and it is difficult to homologise the different parts across species. For example, the large spiny basal flap in *Perochaeta orientalis* (red arrow, Fig. 1H) might be homologous to the unlabelled flap in *Archisepsis* Silva, 1993 (red arrow, Fig. 7) but more species need to be studied before this hypothesis can be supported. This problem is not limited to Sepsidae: In his taxonomic review of the kelp fly family Coelopidae (often used as outgroup for Sepsidae), McAlpine (1991) expressed little confidence in the homology of his proposed phallus terminology, and he only applied descriptive terms. For similar reasons, we here only include SEM images for the *Perochaeta orientalis* phallus as ‘anticipatory’ data for a character system that will only become fully available in the future.

### Costs and benefits of data-rich descriptions

We here richly illustrate the morphology of *Perochaeta orientalis* with line drawings, photography and SEM images. This may raise the question whether too much effort was invested into a single species. However, all these visual data were acquired within a day, while much more time was needed for getting access to the original type material, literature, and confirming species identity. Of course, one obvious question raised by our proposal is where to stop. While we have covered the external morphology with images, our treatment is far from exhaustive. For example, we did not investigate internal morphology, nor the cuticular hydrocarbon profile (Kather and Martin 2012) and UV reflectance (Shevtsova et al. 2011), etc. We could have also added light-transmission photographs of the phallus which would have distinguished sclerotized and membranous parts. Furthermore, we only imaged one specimen of each sex (in addition to the holotype), which may not represent the intra-specific variability. The amount of data to be presented in a description is ultimately up to the author and determined by the tradeoffs between the costs of acquiring additional information and its potential use. Within the last decade we have seen rapid advancements in digital photography and decreases in the cost of acquiring and publishing imaging data. This has led to the much more widespread use of photographs in taxonomic manuscripts. However, we argue the focus has been too much on illustrating those structures that are already known to be important. Let us be more visionary by illustrating even more structures in anticipation of future needs. This does not only apply to new species, but also to species whose descriptions have become inadequate.

One may argue that this will add to the taxonomic impediment, because future descriptions would require more images. This is a legitimate concern, given that taxonomists are already overwhelmed with the amount of undescribed species (Riedel et al. 2013). However, currently much time is invested in long texts which are often of limited value while descriptions that are rich in illustrations can be generated in relatively short amounts of time. For example, with proper equipment, staff can produce 40 high-quality images per day (Tegelberg et al. 2012). Descriptions can then be prepared quickly (e.g., as high-resolution images displayed on a computer screen). Moreover, moving toward descriptions with more images is also an investment into the future. Most taxonomists will concede that it is the processing of inadequate descriptions that are a major reason for the taxonomic impediment. For example, in our case, the main bottle neck in identifying *Perochaeta orientalis* was overcoming issues created by previous taxonomic work. These problems could have been avoided if a re-description had been available or the holotype had been properly illustrated at the time of description.

## Supplementary Material

XML Treatment for Perochaeta orientalis

## Appendix

Scan of precedent descriptions of *Perochaeta orientalis* (doi: 10.3897/zookeys.355.6013.app). File format: Adobe PDF file (pdf).

**Explanation note:** Scanned original and subsequent description of *Perochaeta orientalis* by de Meijere (1913) and Duda (1926). All media have been archived at:

1) Sepsidnet (http://sepsidnet-rmbr.nus.edu.sg/Perochaeta_orientalis.html)

2) Morphobank (http://morphobank.org/permalink/?P1062)
