# Supplementary material for: Using seemingly unnecessary illustrations to improve the diagnostic usefulness of descriptions in taxonomy–a case study on Perochaeta orientalis (Diptera, Sepsidae)
Source: Zookeys. 2013 Nov 25;(355):9–27. doi: 10.3897/zookeys.355.6013 (PMC3867187; doi:10.3897/zookeys.355.6013)
Supplement: Supplementary file 3 — Scan of precedent descriptions of Perochaeta orientalis (doi: 10.3897/zookeys.355.6013.app). File format: Adobe PDF file (pdf). [file ZooKeys-355-009-s001.pdf]

APPENDIX 1:
Scanned original and subsequent description of *Perochaeta orientalis* by de Meijere (1913) and Duda (1926)

A) Scan of original description extracted from:
Meijere, J.C.H. de (1913) H. Sauter's Formosa Ausbeute. Sepsinae. (Dipt.). *Annales historico-naturales Musei nationalis hungarici*, 11: p123.

Nemopoda Rob. Desv.

Nemopoda orientalis n. sp.

Chip-Chip, März, 1 ♂.

Stirn glänzendschwarz, Fühler dunkelbraun; Untergesicht weiss bestäubt. Rüssel dunkelbraun mit gelben Labellen. Thorax bronzefarbig, nur hinten mit zwei kurzen Dorsocentralborsten, auch die zwei Schildchenborsten kurz. Brustseiten glänzendschwarz, Sternopleuren hinten breit weissbestäubt, vorn nur der obere Saum. Hinterleib sehr glänzend bronzefarbig, stellenweise ins Purpurne ziehend, nackt, der zweite Ring merkbar verdickt. Hypopyg mässig gross, glänzendschwarz, an der Wurzel bräunlich, von der Seite gesehen dreieckig, mit einigen Haarborsten, vorn mit zwei nach vorn gerichteten kurzen Fortsätzen, welche etwas gebogen und in der Wurzelhälfte etwas verbreitert sind. Vierter Ring am Bauche vorgezogen,. mit zwei langen gebogenen Fäden. Beine rotgelb, die Wurzel der hinteren Schienen und die Spitze der Tarsen verdunkelt; Hinterschenkel vor der Spitze mit einem schwachen dunklen Ring. Die Beine sind fast nackt, unbeborstet, auch die Vorderbeine ganz unbewaffnet; an der Hinterschiene findet sich auf $^{2}/_{3}$ der Aussenseite ein flaches Höckerchen. Flügel glashell, dritte und vierte Längsader parallel; kleine Querader deutlich jenseits der Mitte der Discoidalzelle. Schwinger gelb mit schwarzem Stiel. Körper- und Flügellänge 4 mm.

Es liegen mir noch drei Weibchen vor (2 von Polisha, Dezember, 1 von Chip-Chip, März), welche den vorhergehenden Männchen sehr ähnlich sehen; wegen einiger kleinen Differenzen zögere ich ein wenig, sie als das zugehörige Weibchen zu betrachten. Der Rüssel ist bei ihnen in grösserer Ausdehnung gelb; die Borsten des Thoraxrückens sind etwas länger, die Sternopleuren sind nur hinten weiss bestäubt, vorn auch am oberen Rande glänzend. die Pteropleuren sind hinten weiss bestäubt (bei *N. orientalis* ♂ ganz glänzend), an den Hinterschenkeln kommt kein dunkler Ring vor; die kleine Querader liegt etwas weiter distalwärts. Weil in Chip-Chip beide Formen erbeutet wurden, ist demnach die Zugehörigk-it wahrscheinlich.

B) Scan of subsequent description extracted from:
Duda, O. (1926) Monographie der Sepsiden. (Dipt.). II. *Annalen des Naturhistorischen Museums in Wien*, 40: pp52-54 (Illustration from Table 4, p109)

8. **Perochaeta orientalis** de Meijere (92) *(Nemopoda)*.

Körperlänge 4 *mm*; Kopf etwas länger als hoch; Gesicht schmutzigbraun, dicht weiß bestäubt; Kiel fast senkrecht abfallend, nicht nasenförmig, sondern schon oberhalb der Backen sanft zum Mundrande zurückweichend; Prälabrum bandartig, schwarz gesäumt; Fühlergruben sehr flach; Stirn glänzend stahlblau, vorn sehr schmal bzw. knapp halb so breit wie bis zum vorderen Punktauge lang; Oz. relativ klein und dünn, wenig über ein Drittel so lang wie ihr Abstand vom Stirnvorderrande; V. kräftiger, aber wenig länger; Pv. ebenfalls kurz, nur etwa halb so lang wie ihr Abstand von den V.; Po. und Orb. fehlend; Scheitel abgeflacht bzw. nicht stärker gewölbt als Stirn und Hinterkopf; dieser schwarz, zart grau reifartig behaart; Augen groß, fast kreisrund, bis an die Gesichtsränder reichend; Backen schmutzig rotbraun, sehr schmal, an schmalster Stelle fast linear, weiß bereift, nicht so weit nach vorn reichend wie die Augen; Vibrissen winzig, doch ist je eine Vibrisse etwa dreimal so lang wie die sehr feinen und kurzen hinteren Oralen, zu denen eine zweite schwache Vibrisse überleitet; Prälabrum schmal, bandartig, schwarz gesäumt, Mundkegel schwarz, Labellen braun; Taster versteckt; Fühler rotbraun, drittes Glied so nur am Grunde, sonst schwarz und schwarz behaart; Arista schwarz, am Grunde wenig verdickt.

Thorax glänzend schwarz; Mesonotum dicht, fein, reifartig, braun behaart, einwärts der Schulterbeulen und der Notopleuralkanten zart weißlich bereift; Schulterbeulen etwas abgeflacht, glänzend schwarz; Akrostichalen winzig, schwarz; a. Mi. zweireihig, d. Mi. einreihig, nur auf der hinteren Mesonotumhälfte deutlich; nur e i n Paar schwacher und kurzer D. vorhanden, H. fehlend, Np. etwas kürzer als die h. Np., auch diese wenig über halb so lang wie ihr Abstand von der v. Np.; Sa. vorhanden, aber sehr klein; Schildchen doppelt so breit wie lang, schwarz, grau bereift, am Grunde sammetschwarz; a. Rb. wenig länger als das Schildchen, l. Rb. fein und kurz, haarig; Meso- und Pteropleuren glänzend schwarz, erstere fein zerstreut behaart; Mp. vorhanden, aber schwach; Sternopleuren nur am Vorderrande glänzend schwarz, sonst dicht weißgrau bestäubt; Mesophragma zentral glänzend schwarz, lateral grau bereift; Schüppchen dunkel, mit schmutzig hellbraunem Rande, dunkel behaart; Schwinger gelb, mit schwarzbraunem Stiel.

Hinterleib glatt, glänzend, schwarz mit violettem Schimmer, hinter dem hinten aufgetriebenen zweiten Tergit deutlich eingeschnürt; Ma. fehlend, dagegen sind zwei lange kräftige Analborsten vorhanden; erstes und zweites Tergit so lang wie das dritte, dieses so lang wie das vierte; dieses etwas länger als das fünfte und sechste Tergit zusammen; Hinterbacken (Fig. 58) hinten mit je zwei langen aufgerichteten Borstenhaaren; Zangen lang und dünn, die Richtung der Hinterbacken fortsetzend, einwärts gekrümmt, bei vollständiger Kreuzung wagerecht abstehend, innen und außen sehr fein zerstreut behaart. Am Bauche sieht man unter dem vierten Tergit je einen kräftigen, den Genitalanhängen von *Themira* analogen langen Fortsatz, dessen kurzes knopfartig verdicktes Endglied an der Innenseite kurz borstig behaart ist und dem apikal je eine kräftige Geißel ansitzt; dieselbe ist am Grunde bandartig verbreitert und gabelt sich in einen inneren oberen kurzen dornartigen und einen sehr langen, geschwungenen, peitschenartigen Endteil; letzterer besteht aus zwei einander eng anliegenden plumpen Geißelhaaren.

Beine gelb, Mittel- und Hinterschienen oben mehr oder weniger verdunkelt; Vorderhüften mit einer schwachen Präapikalen und einigen feinen Härchen, Mittel- und Hinterhüften fein und kurz behaart; alle Schenkel gleichartig, lang, einfach gebildet und nur sehr fein und kurz behaart; Schienen fast gerade, von oben nach unten allmählich dicker werdend; Borsten nebst deutlichen Präapikalen fehlend; Hinterschienen außen hinten etwa am unteren Drittel mit einem kleinen flachen Höcker; Tarsen sehr schlank; Fersen etwa so lang wie die Tarsenreste; Mittelfersen vorn innen sehr dicht, kammartig eng, gleichmäßig kurz-, doch etwas länger als an der Hinterseite und länger als die Vorder- und Hinterfersen behaart.

Flügel farblos, nur in der Costalzelle wie gewöhnlich gebräunt; Costalborsten kurz; Costa vom zweiten Abschnitt ab schwarz, die übrigen Adern hell- bis dunkelbraun; zweiter Costalabschnitt länger als der erste und etwa $2\frac{1}{2}$ mal so lang wie der dritte, dieser doppelt so lang wie der vierte; zweite Längsader nach zunächst geradem Verlauf am Ende vorn weithin konvex zurückgebogen und sehr spitzwinklig in die Costa mündend; Endabschnitt der dritten Längsader vorn konvex und zur schwach S-förmig gebogenen vierten Längsader eine Spur konvergent; mittlere Querader am äußeren dritten Fünftel der Diskoidalzelle; diese hinter ihr relativ breit; Queraderabstand etwa $1\frac{1}{2}$ mal so lang wie die hintere Querader, so lang wie der Endabschnitt der fünften Längsader; hintere Basal- und Analzelle sehr schmal und lang, außen nicht verbreitert; Analader fast den Flügelrand erreichend; Alula abgeflacht, sehr kurz bzw. kürzer als ihre lange Randbehaarung.

Das ♂ ist durch die eigenartig gebildeten Genitalanhänge, die unbeborsteten Vorder- und Mittelschenkel, die lange Stirn, die fehlenden Po., die kurzen Oz., V. und Pv., den kurz beborsteten Thorax, die lange zweite Längsader, die kurze Alula usw. von den *Nemopoda*arten so verschieden, daß sie mir die Bildung einer besonderen Gattung zu erfordern scheint. Ich nenne sie Perochaeta (πηρός = verkrüppelt und χαίτη = Haar).

Zwei ♀♀ des Budapester Museums aus Polisha und Chip-Chip sowie ein ♀ de Meijere's aus Polisha, alle von de Meijere als *orientalis* bestimmt, sind weiter nichts als *bicolor* Wiedem. var. *javanica* de Meijere. Das einzige vorhandene ♂ von *Nemopoda orientalis* de Meijere im Budapester Museum ist bezettelt „Formosa Sauter, Chip-Chip 909. III. *Nemopoda orientalis* det de Meijere. Type".

58. *Perochaeta orientalis* de Meij., Hypopyg.

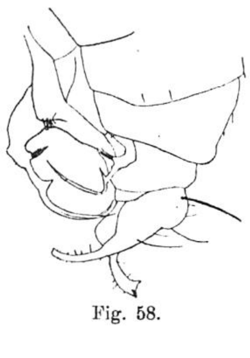

Fig. 58.
